# Inter-trial phase coherence of visually evoked postural responses in virtual reality

**DOI:** 10.1007/s00221-020-05782-2

**Published:** 2020-04-01

**Authors:** David Engel, Adrian Schütz, Milosz Krala, Jakob C. B. Schwenk, Adam P. Morris, Frank Bremmer

**Affiliations:** 1 Department of Neurophysics, University of Marburg, Karl-v.-Frisch-Str. 8a, 35043 Marburg, Germany; 2 Center for Mind, Brain and Behavior (CMBB), University of Marburg and Justus-Liebig-University, Gießen, Germany; 3 Department of Physiology, Neuroscience Program, Biomedicine Discovery Institute, Monash University, Clayton, VIC Australia

**Keywords:** Body sway, Virtual reality, COP, Postural response, Frequency, Phase coherence

## Abstract

Vision plays a central role in maintaining balance. When humans perceive their body as moving, they trigger counter movements. This results in body sway, which has typically been investigated by measuring the body’s center of pressure (COP). Here, we aimed to induce visually evoked postural responses (VEPR) by simulating self-motion in virtual reality (VR) using a sinusoidally oscillating “moving room” paradigm. Ten healthy subjects participated in the experiment. Stimulation consisted of a 3D-cloud of random dots, presented through a VR headset, which oscillated sinusoidally in the anterior–posterior direction at different frequencies. We used a force platform to measure subjects’ COP over time and quantified the resulting trajectory by wavelet analyses including inter-trial phase coherence (ITPC). Subjects exhibited significant coupling of their COP to the respective stimulus. Even when spectral analysis of postural sway showed only small responses in the expected frequency bands (power), ITPC revealed an almost constant strength of coupling to the stimulus within but also across subjects and presented frequencies. Remarkably, ITPC even revealed a strong phase coupling to stimulation at 1.5 Hz, which exceeds the frequency range that has generally been attributed to the coupling of human postural sway to an oscillatory visual scenery. These findings suggest phase-locking to be an essential feature of visuomotor control.

## Introduction

Despite its apparent naturalness, bipedal upright standing is inherently unstable and involves a myriad of complex underlying neural and biomechanical control processes (Peterka 2002; Horak 2006). Understanding how humans control their balance and posture can benefit many applications such as diagnosing and preventing disease, as well as accelerating rehabilitation (Horak 2006). We manage to maintain our upright stance via a complex control system, which, on the sensory side, is primarily based on an interplay between vision, vestibular information and proprioception (Bronstein et al. 1990; Horak and Macpherson 1996; Horak 2006; Mahboobin et al. 2009; Bronstein 2019). Weighting across these modalities is highly dynamic and depends on factors such as availability and variability of signals. However, for many situations in our everyday behavior, vision has a dominant role, and visual clues alone strongly influence our balance (Berthoz et al. 1975, 1979; Soechting and Berthoz 1979; Bronstein 1986; Wade and Jones 1997; Laurens et al. 2010).

Stable balance is mainly achieved by controlling the body’s center of mass (COM) and keeping it within a distinct area of stability which physically supports the upright human body (Horak and Macpherson 1996; Scholz et al. 2007, 2012; Sousa et al. 2012). Locating the COM within the complex mass distribution of a human body is a difficult endeavor, which is why foot center of pressure (COP) is widely used to investigate postural sway and whole-body movements (Winter 1995; Winter et al. 1996). In terms of physics, COP describes the accumulation of all forces the human body enacts on the ground on one spot, which can be measured via the reactive ground forces using a force platform. For small angular displacements, the COP time-course is closely related to that of COM, making it a valid measure to investigate postural control (Winter et al. 1996; Yamamoto et al. 2015).

Because vision is so vital to the process of maintaining balance, understanding the way in which visual information and its processing influence posture and balance control is crucial. As early as in the 1970s, it has been shown that moving visual scenes lead to postural readjustments during quiet standing (Lee and Lishman 1975; Lestienne et al. 1977, van Asten et al. 1988a, b). Bronstein (1986) called this effect *visually evoked postural response* (VEPR). In this context, a visual stimulation that is often used is the “moving room” paradigm (Lee and Aronson 1974; Lee and Lishman 1975; Schöner 1991), during which a real or virtual room is moved relative to the subject, eliciting the illusion of self-motion and subsequent postural responses. Despite the numerous variations of experimental designs carried out within the field, it is generally concluded that certain characteristics of a moving visual stimulus affect postural sway. These include structure (van Asten et al. 1988a; Tossavainen et al. 2003), velocity (Stoffregen 1986; Kuno 1999; Barela et al. 2009; Dokka et al. 2009; Day et al. 2016; Holten et al. 2016) and predictability (Guerraz et al. 2001; Musolino et al. 2006; Barela et al. 2009). For periodical stimuli in particular, the effect on posture and balance depends on the frequency (Dijkstra et al. 1994; Oida et al. 1995; Jeka et al. 1998; Kuno et al. 1999; Scholz et al. 2012; Hanssens et al. 2013) and amplitude (Peterka and Benolken 1995; Peterka 2002; O’Connor et al. 2008) of the modulation.

We regularly encounter oscillating visual motion in our natural behavior, for example during walking, which is why our visual system is highly sensitive to this kind of stimulus. Because of their simple implementation and analytically beneficial properties, periodical perturbations of the visual scene in the form of sinusoidal oscillations have become a common method of assessing the influence of vision on posture (e.g. Lee and Lishman 1975; Dijkstra et al. 1994; Peterka and Benolken 1995; Keshner and Kenyon 2000; Loughlin and Redfern 2001; Oie et al. 2002; Musolino et al. 2006; Redfern et al. 2007; Scholz et al. 2012; Hanssens et al. 2013; Cruz et al. 2018). Being performed in such a way, these studies allow for insights into the adjustments of the postural control system to an ongoing stimulation over longer periods of time. Periodical motion of the visual scene can be regarded as a stationary state, and the coupling to this motion can therefore be considered a measurement of stability (Schöner 1991). In these scenarios, the balance control system tries to achieve a stationary state within the recurring movement. This is referred to as dynamic equilibrium (Horak and Macpherson 1996), a steady-state coupling of the balance control system to an ongoing visual perturbation (Peterka 2002). According to van Asten et al. (1988b), visual motion needs to occur in frequencies below 0.5 Hz in order to induce coherent body sway, i.e. consistent sway patterns which can be attributed to the movement of the visual surround. Low frequencies (0.1 to 0.3 Hz) typically have the largest effect on postural sway (Lestienne et al. 1977; van Asten et al. 1988b; Schöner 1991; Masson et al. 1995; Kiemel et al. 2006; Hanssens et al. 2013).

A common method used to investigate the strength of coupling to a periodical visual stimulus is to compute frequency spectrograms of the responses and analyze these according to the power of the frequency contained in the stimulation (i.e. to quantify the transfer function of the system). These spectrograms reveal the extent to which the frequency components of the visual motion stimulus can be found in the bodily response of the subject. This gives insight into how the postural control system adapts to a constantly perturbed visual input and achieves a steady state. In doing so however, a key challenge is that postural spectrograms typically show high inter-subject variability, with many subjects (up to half according to Kay and Warren 2001) showing weak or no effects of the visual stimulus (Dijkstra et al. 1994; Kay and Warren 2001; Peterka 2002; Sparto et al. 2004; Mahboobin et al. 2005). Sparto and colleagues (2004) noted the need for a method to quantify the statistical significance of a subject’s postural spectrogram as response to a particular stimulus.

Taken together, there is demand for a comparable parameter by which to investigate and quantify postural responses to oscillatory stimuli, to gain further insight into visuomotor processing of periodical visual input. Here, we investigated COP responses to sinusoidal visual perturbations in VR. Importantly, we present both an alternative method to quantify the coupling of postural sway to a moving visual surrounding, as well as a bootstrapping approach to test statistical significance.

## Methods

### Participants

Ten human subjects (20–32 years) with normal or corrected to normal vision and no known neurological or musculoskeletal impairments participated in the study. All subjects gave written informed consent prior to the experiment, including that regarding the storing and processing of their data. Research was approved by the Ethics Committee of the Psychology Department, University of Marburg. All research was conducted according to the Declaration of Helsinki.

### Stimulus and apparatus

**Fig. 1:**
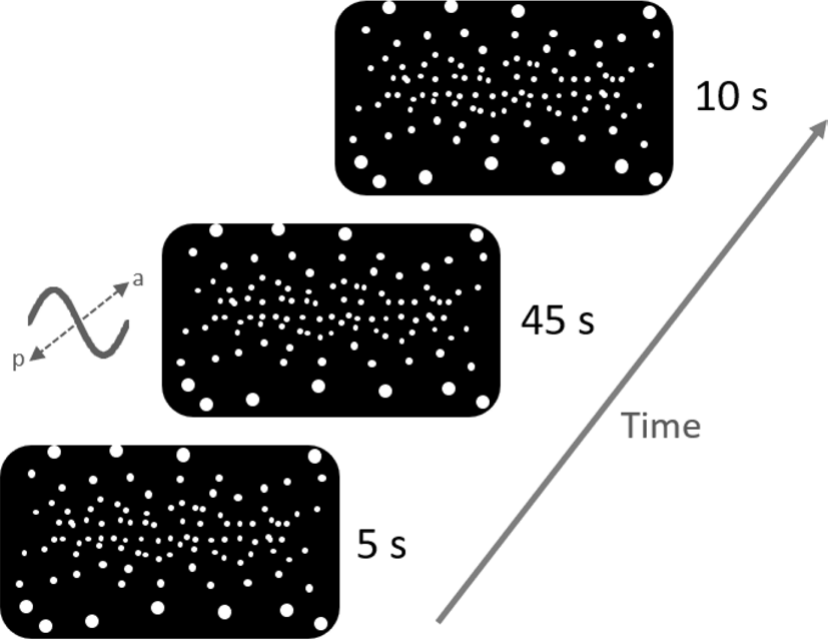
The stimulus consisted of a 3-D cloud of random white spheres on a black background. After 5 s being stationary, the cloud of spheres then started moving sinusoidally for 45 s in the anterior–posterior direction, simulating a movement of the outside world. The movement was followed by another static period lasting for 10 s

Visual stimuli were presented through an Oculus Rift DK2 (Oculus VR, Irvine, CA, USA) head mounted display. Subjects stood on a force plate (Accusway, AMTI, Watertown, MA, USA) to measure their ground reaction forces in order to calculate the trajectory of their COP (2-D) during trials. The sampling rate of the force plate was set to 50 Hz. Subjects were told to stand relaxed with their feet about hip width apart and parallel on the ground. Their arms were to hang loosely at the side of their body, and they were instructed to maintain their gaze straight ahead. For safety reasons, subjects wore a harness during visual presentation, which was not providing lift during trials, so as not to interfere with the subject’s natural posture and balance. The stimulus was created using the *PsychVRToolbox* in MATLAB (The MathWorks, Inc., Massachusetts, USA), based on an OpenGL framework. It consisted of a 3-D cloud of white spheres on a black background distributed randomly within a virtual space centered around the observer’s head. The span of this space was 200 m, 150 m, and 200 m in the left-to-right, up-to-down, and front-to-back axis, respectively, yielding a sphere density of ${8\times 10}^{-4}$/${\mathrm{m}}^{3}$. Spheres within 10 m and beyond 100 m from the observer were not rendered. The size of the spheres scaled with distance, with *glPointSize* set to 15. Field of view was 90° horizontally and 110° vertically. The framerate of the headset was 75 fps. The cloud remained static for 5 s, followed by a 45 s sinusoidal movement of the cloud along the anterior–posterior axis with a simulated peak-to-peak amplitude of 1.5 m. We chose this large amplitude, since pilot recordings had shown strong postural responses of our subjects in the context of this stimulus. The movement was subsequently followed by another 10 s static dot cloud to provide the subjects with relaxation time (Fig. 1). Every subject performed 20 trials in random order for each of five different stimulus frequencies (conditions). Out of those frequencies, three were determined in a preceding experiment individually for each subject. They ranged from 0.3 Hz to 1.3 Hz and their average was 0.40 ± 0.05 Hz, 0.58 ± 0.09 Hz and 0.70 ± 0.1 Hz (mean ± stem). The remaining two frequencies constituted low and high control frequencies at 0.2 Hz and 1.5 Hz respectively and were presented to all subjects. All our analyses are based on these latter two frequencies, with only the bootstrapped based computation of baseline body sway (see “Statistical analysis—background simulation”) to also include the remaining three.

### Data analysis

#### Wavelet decomposition

An important approach to investigate time-series data is to obtain a frequency-domain representation of the data by decomposing the time-domain signal into its frequency content. This is commonly achieved using a Fast-Fourier-Transform (FFT), which fits a composition of sinewaves of different frequencies and phases to the signal and thus reveals information about the power and phase of each frequency present in that signal. However, Fourier-analysis comes with two major limitations: It generally assumes stationarity of the signal and makes it difficult to visualize changes in frequency-structure over time. The individual sine waves used for FFT have no temporal localization. It is possible to extract small time windows of the signal and perform FFT on those to investigate temporal changes. However, this also comes with limitations, especially in regard to low frequencies like those employed in our study, as they require a minimum time series length. Even though the stimulus used in the study was stationary over time, i.e. a single sinusoid, a constant power of specific frequencies in the COP responses could not be assumed. Together with the finite nature of the recorded signals, this leads potentially to artifacts in the decomposition when using FFT, because the signals can’t accurately be reconstructed using indefinite sinewaves. These artifacts become enhanced by the subsequent ITPC analysis (see “Inter-trial phase coherence”), making it hard if not impossible to interpret when based on FFT decomposition.

These considerations motivated us to employ time-resolved frequency decomposition methods, one of which is wavelet decomposition. Broadly, a wavelet consists of a temporally localized wave at a distinct frequency with finite time duration, which is shifted along the time-signal and convoluted with this signal at each shift. Using wavelets of different frequencies and durations, this results in a time-resolved frequency-phase-representation of the signal (Abate et al. 2002; Cohen 2014).

The unfiltered time course of a subject’s COP trajectory in each trial underwent wavelet analysis based on a “Morlet”-Wavelet (Cohen 2019). This was chosen due to its analytical nature, which allows for a simple conversion from the wavelet scale to a single frequency, as well as due to its similarity to the sinusoidal structure of the Morlet wavelet family. The analysis yielded complex time–frequency resolved wavelet coefficients ${W}_{(f, t)}$ revealing the content of COP responses in regard to frequency power and phase. In addition, the resulting wavelet spectra were averaged over time to gain insight into the global power distribution across frequencies.

#### Inter-trial phase coherence

Phase coherence, in general, constitutes a measure of temporal synchronicity between a set of oscillations. In EEG-related studies, a phase-lock value to determine synchronization between two neuroelectric signals was introduced by Lachaux et al. (1999) and subsequently led to intertrial phase coherence (ITPC) being established as a method to investigate phase synchronicity between trials (Cohen 2014; van Diepen and Mazaheri 2018). Analogously, we used the data achieved through the wavelet analysis to investigate phase relationships of all frequency bands across trials for each subject and condition. To calculate the ITPC of the postural responses, we divided the absolute value of the mean amplitudes of the wavelet coefficients across trials by the mean of absolute values of the amplitudes (1). The numerator is phase-sensitive, whereas the denominator is not.

1 $$\psi \left(f,t\right)= \frac{\left|{\langle W(f,t)\rangle }_{m}\right|}{{\langle \left|W(f,t)\right|\rangle }_{m}}$$

$\psi$: phase coherence coefficient, $W$: wavelet coefficient, $f$: frequency, $t$: time, $m$: trial.

This quotient results in phase coherence coefficients $\psi$, a measure of relative phase (i.e. phase synchronicity) between trials for each frequency band at each point in time. These coefficients take a value between 0 (entirely incoherent phases) and 1 (entirely coherent phases). Within this context, a high value close to 1 means that on average, trials of the same condition (i.e. stimulus frequency) display similar phases at the same frequency and point in time ($f$ and $t$ in (1), respectively).

### Statistical analysis—background simulation

In order to verify that a subject’s response is causally linked to the stimulus, the response to a given stimulus frequency must be tested against a baseline condition or null distribution. As our experimental paradigm lacked an explicit baseline, we calculated a null distribution for the wavelet and the subsequent phase coherence spectra. For a given stimulus frequency, we constructed a bootstrapped data set out of the trials from all other conditions; that is, those of other stimulus frequencies, which varied across subjects. Each other condition that went into the bootstrapping had the same number of trials (20) as the tested frequency. A true baseline would consist of a scene with a similar degree of visual stability/disturbance which does not contain coherent anterior–posterior motion at the tested frequency. In this way, if the tested condition was different from such a baseline, this difference could be attributed to the presence of coherent visual motion at the given frequency and not to a general disturbance in visually guided postural stabilization within the given setup.

Using the *bootci* function in MATLAB (The MathWorks, Inc., Massachusetts, USA), 1000 random trial sets of equal size were drawn with replacement and their wavelet and ITPC spectra were calculated. This yielded distributions of the resulting power and phase-coherence values, respectively, of which 95% confidence intervals could be determined for each frequency in the COP spectrogram. In this way, the resulting distributions of power and phase-coherence values represent a range of average subject behavior when the visual scene does not contain the tested frequency. This generated a reliable background for responses of each individual subject across all frequencies and points in time. We considered values of wavelet power or ITPC in the original data set that were above the upper 95% border of the background to be statistically significant.

To avoid false positives, we applied a correction for multiple comparisons according to Guthrie and Buchwald (1991). Consecutive samples in the frequency spectra might not be statistically independent. Hence, the first-order autocorrelation of the difference between each response spectrum and the respective bootstrapped background was taken into account. We acquired the autocorrelation coefficients of sections consisting of *T* = 50 samples which were centered around each investigated frequency in the respective spectrum. These coefficients yielded critical numbers of consecutive samples (${n}_{\mathrm{c}\mathrm{r}\mathrm{i}\mathrm{t}}$) with a *p* < 0.05, which needed to exceed the background in order for the observed part of the spectrum to be considered statistically significant (see Guthrie and Buchwald 1991). Eventually, in each case, the number of actual samples exceeding the background ${n}_{\mathrm{s}\mathrm{a}\mathrm{m}\mathrm{p}\mathrm{l}\mathrm{e}}$ was determined and compared against ${n}_{\mathrm{c}\mathrm{r}\mathrm{i}\mathrm{t}}$.

### Comparison of methods—inter-subject variability.

To gain insight into further functional characteristics of ITPC, we compared the wavelet-based spectral analysis and corresponding ITPC according to inter-subject variability. For each subject, we averaged the wavelet power and ITPC spectra over time and picked the values of the frequency bins closest associated with the stimulation frequencies at 0.2 Hz and 1.5 Hz, respectively. Subsequently, we investigated the raw as well as mean-normalized distributions of these values at both conditions for each of the two methods.

## Results

One of the subjects reported vertigo and dizziness during the experiment and hence was excluded. We investigated subjects’ COP responses to pure sinusoidal anterior–posterior visual oscillations in VR at a low (0.2 Hz) and a high frequency (1.5 Hz). Wavelet decomposition of the COP signals yielded time–frequency resolved power spectra, which revealed the extent to which each frequency was present in the postural response at each point in time. Subsequently, we calculated the time–frequency resolved ITPC, which gave insight into how stable the phase of the postural response in a given frequency band remained across trials. In order to test the significance of the responses for each subject and condition, we created a bootstrapped background of average frequency content of the COP signals from all the trials that did not contain the stimulus frequency of that condition. Lastly, since inter-subject comparability marks a significant challenge in the field, we compared power and ITPC spectral analyses according to the inter-subject variance of their results.

### Single subject data

**Fig. 2:**
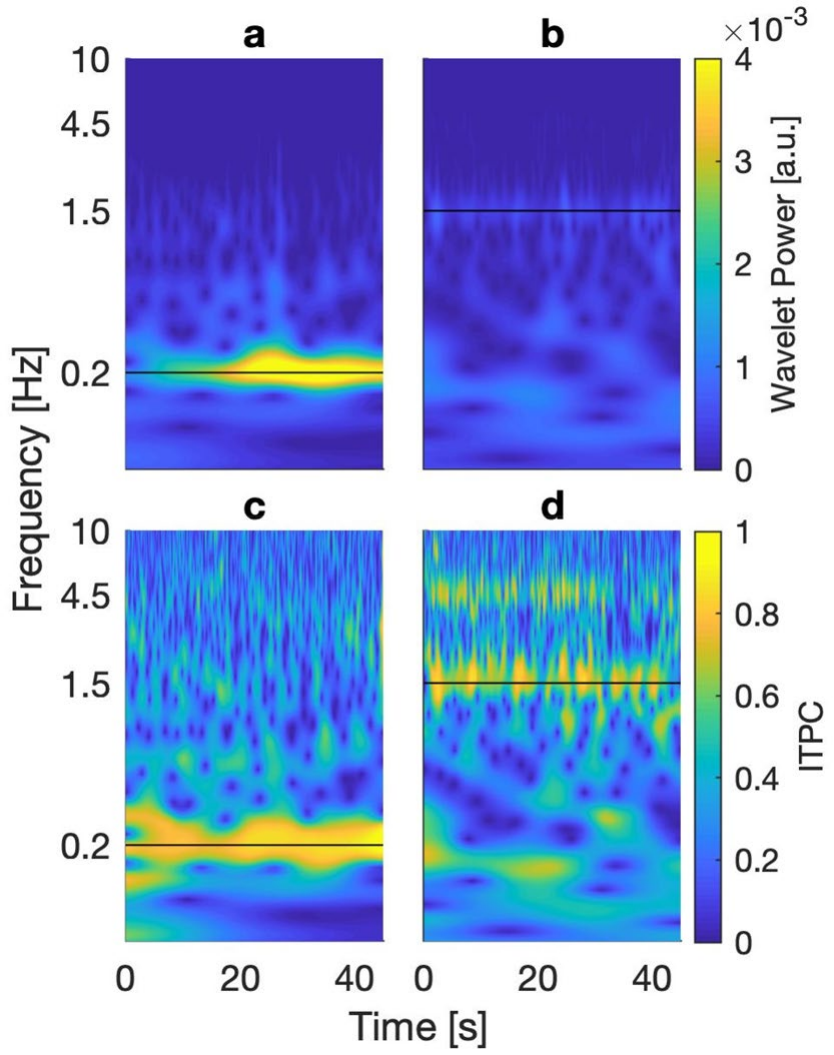
Time–frequency resolved spectra of COP signals obtained from subject EO, averaged over 20 trials. Black horizontal bars represent frequency of visual stimulation. **a** Wavelet power spectrum for visual stimulation at 0.2 Hz. **b** Wavelet power spectrum from visual stimulation at 1.5 Hz. **c** ITPC spectrum from stimulation at 0.2 Hz. **d** ITPC spectrum from stimulation at 1.5 Hz. In contrast to the wavelet power spectrum, ITPC revealed strong coherence for stimulation at both 0.2 and 1.5 Hz, with an additional frequency band of high phase coherence at about triple the frequency of visual stimulation for the 1.5 Hz condition

**Fig.3:**
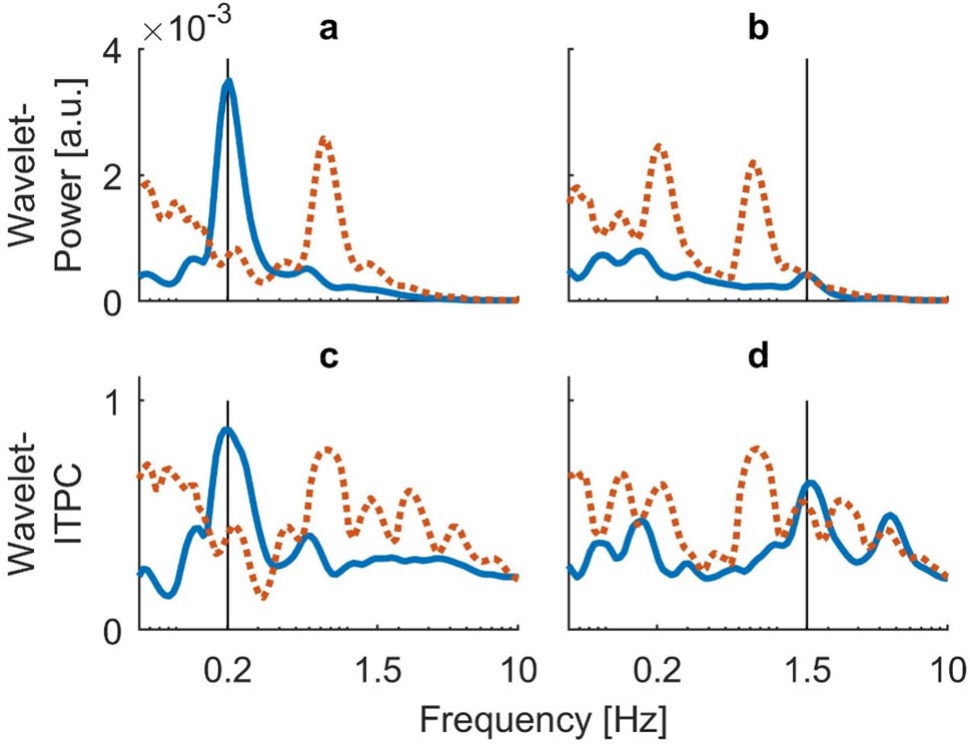
Time-averaged (global) spectra of COP-signals obtained from subject EO. Blue lines indicate mean response over 20 trials. Orange dotted lines represent the calculated upper 95% confidence interval of the bootstrapped background. Black vertical bars represent frequency of visual stimulation. **a** Global wavelet power spectrum for visual stimulation at 0.2 Hz. **b** Global wavelet power spectrum from visual stimulation at 1.5 Hz. **c** Global ITPC spectrum from stimulation at 0.2 Hz. **d** Global ITPC spectrum from stimulation at 1.5 Hz. All spectra show significant responses at the respective stimulus frequency, with ITPC revealing a second significant peak at around 4.5 Hz for stimulation at the high frequency. The remaining frequencies that went into the null distribution for this subject were 0.7 Hz, 1.3 Hz and 1.6 Hz

Figures 2 and 3 show sample data of a representative subject averaged across 20 trials for each stimulation frequency. For visual motion at 0.2 Hz, the wavelet power spectrum yielded a clear response in a frequency band around the stimulated frequency throughout the length of the trials (Fig. 2a). Averaging this power spectrum over time (continuous line) resulted in a clear peak at the stimulus frequency, far exceeding the calculated 95% significance threshold (dashed line, Fig. 3a). This finding was confirmed in the ITPC spectrum, which revealed strong phase synchronicity over the entire time course at the same frequency band (Fig. 2c), indicating a strong and stable coupling to the presented visual perturbation. This result was also reflected in the time-averaged frequency spectrum, at which a clear peak of strong phase synchronicity was visible around the stimulus frequency of 0.2 Hz, clearly above significance threshold (Fig. 3c).

For stimulation at 1.5 Hz, wavelet power only yielded weak responses at the frequency band at which visual stimulation took place. The power spectrum was dominated by lower frequencies in the range of up to 0.4 Hz. This finding was confirmed in the time-averaged spectrum, where there was a small peak around the stimulation frequency, but much more dominant power in lower frequencies. Both of these findings are in line with existing literature. The ITPC-spectrum revealed a clear and stable phase coupling between trials around the stimulated frequency, with the low frequency noise being reduced. Unexpectedly, a second frequency band of coherent phase became apparent throughout nearly the entire duration of visual motion. This frequency band was located at about triple the frequency of the visual input (centered on *f* = 4.6 Hz). The time-averaged frequency spectrum confirmed this finding, showing a peak at the frequency of visual motion, as well as a second peak at about triple the frequency (Fig. 3d). While this characteristic of the postural response was found in seven of our nine participants, two of them exhibited no significant response to the stimulation in both the wavelet power and the ITPC of their COP trajectories.

### Group data

**Fig. 4:**
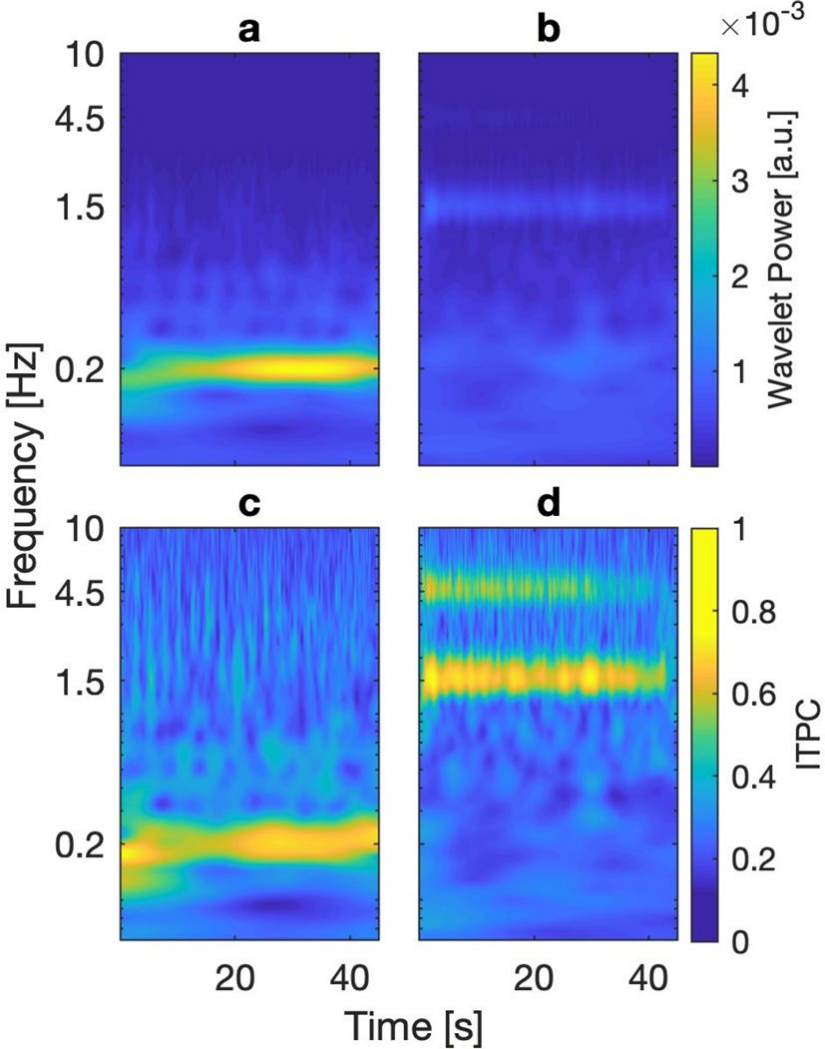
Time–frequency resolved wavelet power and ITPC spectra on group level (*n* = 9). **a** Averaged wavelet power spectrum of the COP signals for visual stimulation at 0.2 Hz. **b** Averaged wavelet power spectrum from visual stimulation at 1.5 Hz. **c** Averaged ITPC spectrum from stimulation at 0.2 Hz. **d** Average ITPC spectrum from stimulation at 1.5 Hz. For stimulation at 1.5 Hz, the resulting ITPC spectrum shows strong synchronicity between trials at the stimulation frequency, eradicating all low-frequency noise presence in the power spectrum, while also revealing a second frequency band of constant phase synchronicity at around triple the frequency of visual stimulation

At the group level (grand average), for stimulation at 0.2 Hz, power analysis of subjects’ COP trajectory during the time of scene movement (45 s) yielded a strong response at the presented oscillatory frequency (Fig. 4a). There was a clear, ongoing steady-state response throughout the course of the trial, dominating the spectrum. For stimulation at 1.5 Hz, a considerably weaker, but still constantly present response was evident in the wavelet spectrum (Fig. 4b). However, in line with the single subject data (Figs. 2b and 3b), for stimulation at this considerably higher frequency the COP trajectory in general shows very strong additional oscillations at the lower end of the spectrum up to about 0.4 Hz. These dominate the overall spectrum as low-frequency noise, and can be attributed to naturally occurring body sway (e.g. Singh et al. 2012). ITPC analysis revealed a clearly visible phase synchronicity between trials at both frequency bands at which visual stimulation occurred. For stimulation at 0.2 Hz, the spectrum yielded a phase coherence between trials only at that frequency band (Fig. 4c), being in line with the result in the power spectrum (Fig. 4a). For stimulation at 1.5 Hz, the ITPC spectrum also revealed a strong phase coherence around the stimulation frequency throughout the course of the visual movement (Fig. 4d). As opposed to the results in the power spectrum (Fig. 4b), the low frequency noise was not apparent in the ITPC spectrum. Analogous to the single subject data (Figs. 2d, 3d), phase coherence at a second frequency band became apparent, almost throughout the entire length of the trials. This phase coherence occurred at about triple the fequency of visual motion, i.e. at about 4.5 Hz.

**Fig. 5:**
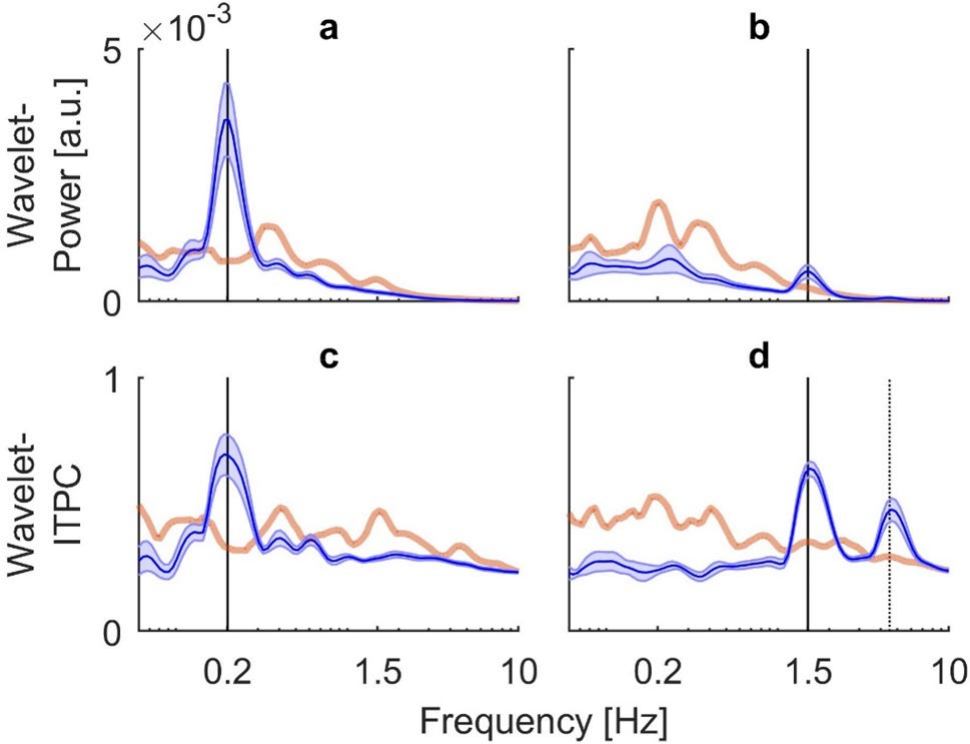
Group results for time-averaged (global) wavelet power and ITPC spectra of the obtained COP signals. Blue lines indicate mean across subjects with shaded areas representing standard error. Orange transparent lines show mean upper 95% confidence boundary of the bootstrapped background. Black vertical bars indicate frequency of visual stimulation. **a** Global wavelet power for visual stimulation at 0.2 Hz. **b** Global wavelet power of stimulation at 1.5 Hz. **c** Global ITPC for stimulation at 0.2 Hz. **d** Global ITPC for stimulation at 1.5 Hz. All spectra show a significant response at the respective stimulus frequency. For stimulation at 1.5 Hz, ITPC reveals an additional significant phase coupling at around triple the frequency, the black dotted line indicates the frequency value of 4.5 Hz

Time-averaging of the power and coherence spectra allowed us to quantitatively analyze power and ITPC coefficients across frequencies for each condition. The results are shown in Fig. 5. In order to correct for multiple comparisons, we calculated the critical number of consecutive samples to exceed the background for each condition and method and compared it to the actual number of samples yielded by the spectra (Guthrie and Buchwald 1991). Due to the high autocorrelation of the spectra, the significance threshold turned out to be ${n}_{\mathrm{c}\mathrm{r}\mathrm{i}\mathrm{t}}$ = 9 in all cases (see “Methods” for details). For stimulation at 0.2 Hz, the global power spectrum revealed a clear peak at the frequency at which visual stimulation occurred. The response far exceeded the significance threshold (${n}_{\mathrm{s}\mathrm{a}\mathrm{m}\mathrm{p}\mathrm{l}\mathrm{e}}$=12) (Fig. 5a). Plotting time-averaged ITPC against frequency for the same stimulation also revealed a strong single peak of phase synchronicity at the frequency band of visual oscillation, which clearly exceeded the significance threshold (${n}_{\mathrm{s}\mathrm{a}\mathrm{m}\mathrm{p}\mathrm{l}\mathrm{e}}$=12) (Fig. 5c). For stimulation at 1.5 Hz, the global power spectrum revealed a distinct, albeit small peak at the stimulus frequency. General power of the noise at lower frequencies dominated the spectrum. Only power in the range of the stimulus frequency exceeded the upper boundary of the background and reached statistical significance (${n}_{\mathrm{s}\mathrm{a}\mathrm{m}\mathrm{p}\mathrm{l}\mathrm{e}}$=9) (Fig. 5b). Time-averaged ITPC at the higher frequency revealed a strong single peak of phase synchronicity at the frequency band of visual oscillation which again clearly exceeded the significance threshold (${n}_{\mathrm{s}\mathrm{a}\mathrm{m}\mathrm{p}\mathrm{l}\mathrm{e}}$=11). Unexpectedly, as could already be seen in the time-resolved spectrum (Fig. 4d), a second significant phase coupling at around 4.5 Hz emerged (${n}_{\mathrm{s}\mathrm{a}\mathrm{m}\mathrm{p}\mathrm{l}\mathrm{e}}$=16) (Fig. 5d).

### Comparison of methods

At the group level, subjects showed a significant response in their body sway to the oscillatory stimulus in the respective frequency band for both low and high frequency conditions. However, responses to the high frequency were weak (Figs. 4b, 5b) and varied strongly between subjects. On the contrary, we found seven out of nine subjects to exhibit a reliable phase coherence in the frequency band around the presented frequency, as opposed to only two subjects when considering frequency power.

**Fig. 6:**
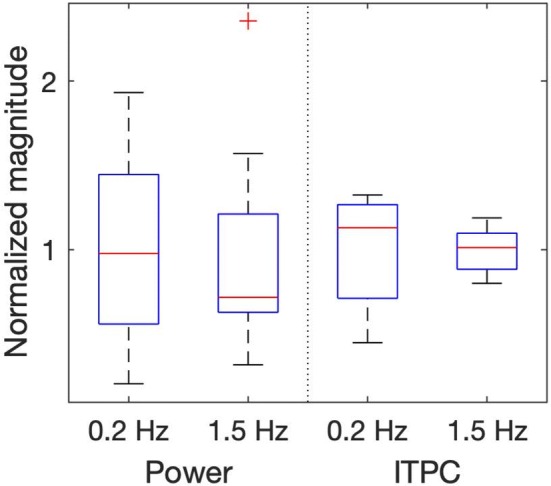
Distribution of mean responses across subjects for both conditions and methods of analysis shown as boxplots. For comparison, results were normalized according to their mean. ITPC shows less variance in responses across subjects than classic wavelet-power analysis for both visual frequencies

**Fig. 7:**
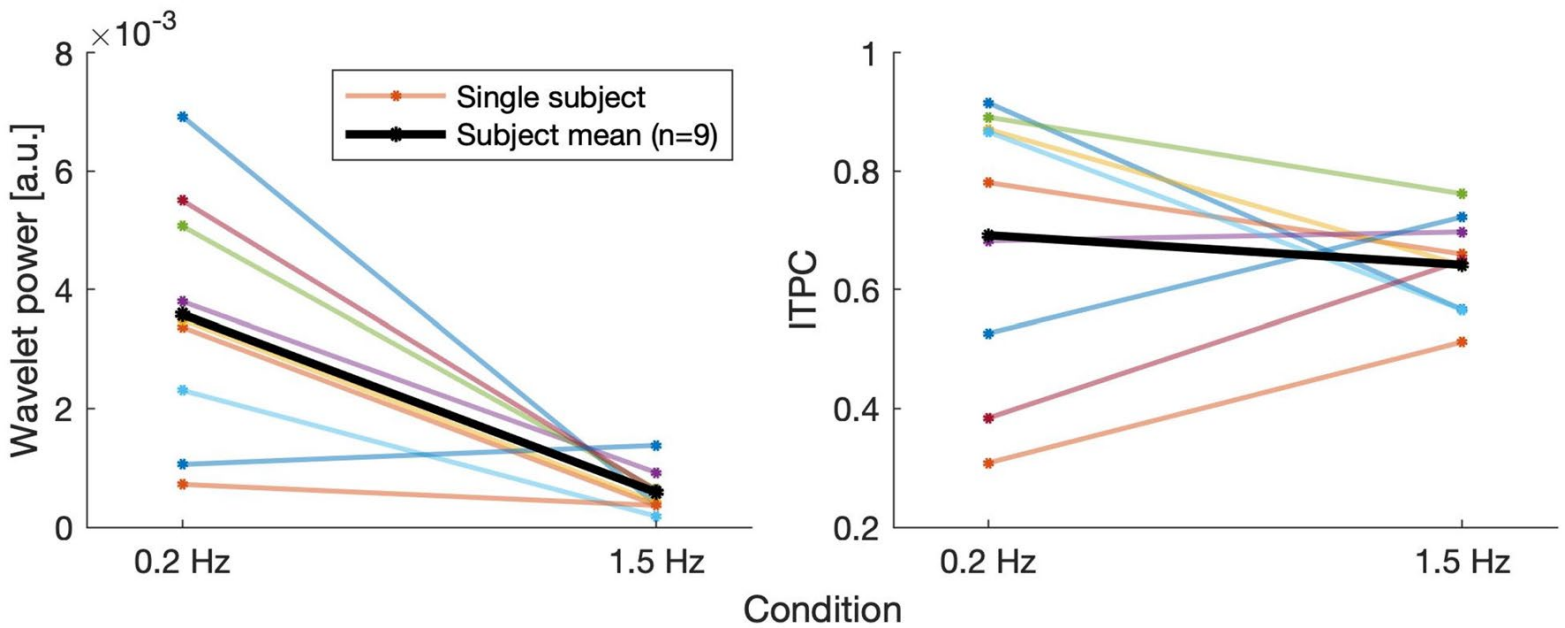
Time-averaged values of wavelet power (left panel) and ITPC (right panel) for both visual conditions. Average values of single subjects across trials in colors, mean across subjects in black. Wavelet power reveals a clear discrepancy in the strength of responses between conditions with significantly weaker responses at the high frequency (*p* < 0.0005). ITPC responses were not significantly different between the two stimulus frequencies (*p* = 0.3401)

Figure 6 shows the distribution of responses across subjects. In the resulting spectra, the average values across time for both methods of analysis were determined for each subject and condition. Since power and ITPC differ in their absolute values, both distributions were normalized by dividing them by their respective mean. This resulted in higher comparability with each distribution having the same relative spread as prior to normalization. Responses at the higher control frequency of 1.5 Hz were generally more precise than responses to oscillation at 0.2 Hz, as they showed less variance. This is mainly due to the logarithmic frequency-scaling of the used wavelet analysis, which results in a naturally lower resolution for higher frequencies. ITPC analysis showed less variance in both frequency bands when compared to pure wavelet power. Figure 7 shows the raw values of the same average responses for both conditions for every subject as well as the mean across subjects. The left panel shows the average values for the wavelet power of subjects’ responses to both visual stimulations. As could already be seen in Fig. 6, variability of responses was high, especially at low frequency stimulation. Also in accordance to Fig. 6, the values for stimulation at 1.5 Hz showed less variance, due to the logarithmic frequency resolution of the analysis, as explained above. Responses for the high frequency stimulation, however, were considerably smaller than for the low frequency stimulation (Wilcoxon rank sum test, *p* < 0.0005). In contrast, ITPC values were not significantly different for the two stimulation frequencies (Wilcoxon rank sum test: *p* = 0.3401).

### Results based on classic FFT decomposition

**Fig. 8:**
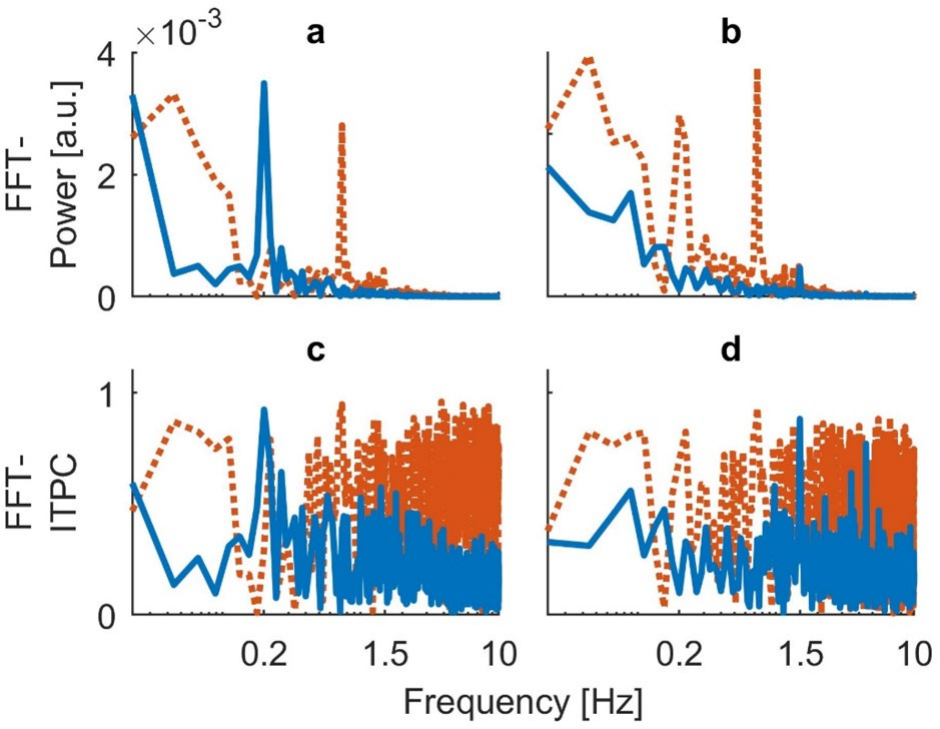
FFT-based spectra obtained from subject EO. Blue lines indicate mean response over 20 trials. Orange dotted lines represent the calculated upper 95% confidence interval of the bootstrapped background. **a** FFT power spectrum for visual stimulation at 0.2 Hz. **b** FFT power spectrum from visual stimulation at 1.5 Hz. **c** FFT-based ITPC spectrum from stimulation at 0.2 Hz. **d** FFT-based ITPC spectrum from stimulation at 1.5 Hz. The spectra reveal the artifacts resulting from FFT decomposition, particularly in the bootstrapped background of ITPC. These include a drift in low frequencies and substantial noise in higher frequencies (**c**, **d**)

**Fig. 9:**
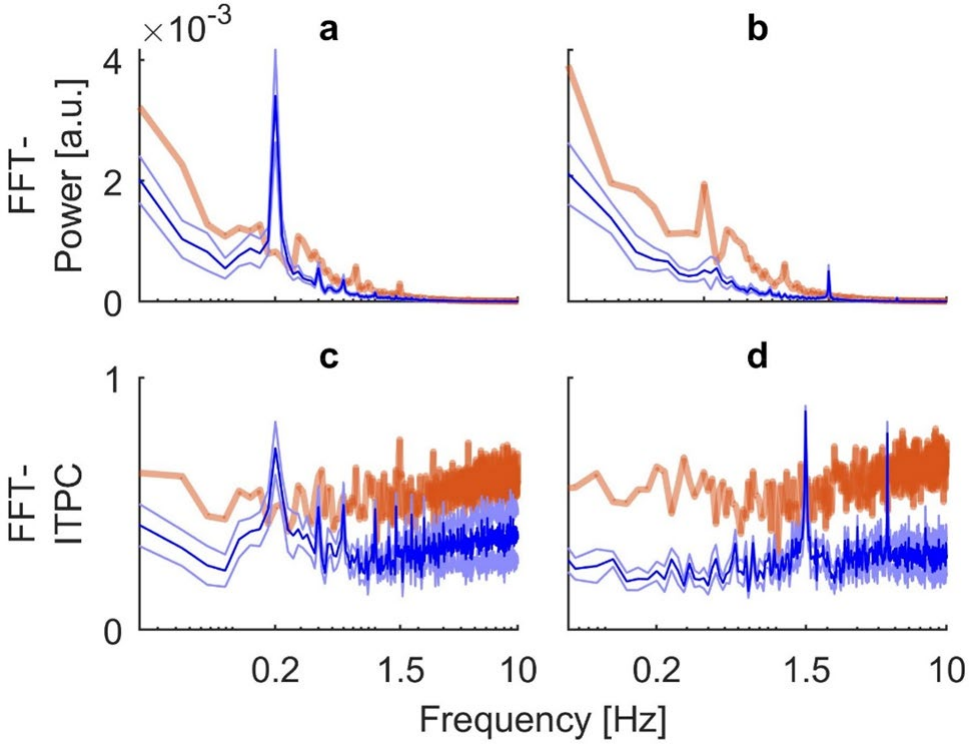
Group results for FFT-based power and ITPC spectra of the obtained COP signals. Blue lines indicate mean across subjects with shaded areas representing standard error. Orange transparent lines show mean upper 95% confidence boundary of the bootstrapped background. **a** FFT power for visual stimulation at 0.2 Hz. **b** FFT power of stimulation at 1.5 Hz. **c** FFT-based ITPC for stimulation at 0.2 Hz. **d** FFT-based ITPC for stimulation at 1.5 Hz

As explained in our methods (Data analysis), we used time-resolved wavelet decomposition to obtain the frequency-representation of our data on which we performed the subsequent ITPC analysis. To illustrate the potential problems associated with a rather classic Fourier-based decomposition, we also calculated the FFT spectra of subjects’ COP trajectories and performed our ITPC analysis on those. The results can be seen in Figs. 8 (single subject) and 9 (group data). First of all, visual inspection of the FFT decomposition confirms the findings of our time-averaged wavelet analyses (Figs. 3 and 5), as the frequency spectra reveal high power in the responses at both stimulated frequencies. In the data obtained from subject EO, there are clearly visible artifacts in the ITPC spectra, especially in the bootstrapped background. These consist of a drift with constant high power in the lower frequencies as well as substantial noise in the higher frequencies (Fig. 8c,d). For the group data, we performed significance testing with correction for multiple comparisons analogous to the wavelet-based spectra. Since the FFT-based spectra had a low auto-correlation, the critical number of consecutive samples to exceed the 95% confidence background was lower than for the wavelets. The threshold ${n}_{\mathrm{c}\mathrm{r}\mathrm{i}\mathrm{t}}$ was 4 for all measures except for ITPC at 4.5 Hz, where ${n}_{\mathrm{c}\mathrm{r}\mathrm{i}\mathrm{t}}$ was 3. For stimulation at 0.2 Hz, the clearly visible peaks in the power and ITPC spectra both reached significance level (${n}_{\mathrm{s}\mathrm{a}\mathrm{m}\mathrm{p}\mathrm{l}\mathrm{e}}$=4) (Fig. 9a, c). For stimulation at 1.5 Hz, the small but distinct peak in the power spectrum (Fig. 9b) was also significant in regard to multiple comparisons (${n}_{\mathrm{s}\mathrm{a}\mathrm{m}\mathrm{p}\mathrm{l}\mathrm{e}}$=4). For ITPC at 1.5 Hz, the peak at the stimulus frequency and its triple at 4.5 Hz were clearly visible in the spectrum (Fig. 9d). However, both of them did not reach significance level when tested for multiple comparisons (${n}_{\mathrm{s}\mathrm{a}\mathrm{m}\mathrm{p}\mathrm{l}\mathrm{e}}$=3 and 2, respectively).

## Discussion

We observed visually evoked postural responses (VEPRs) coupled to sinusoidal moving room-perturbations in VR, consistent with preceding studies employing real world (Lee and Lishman 1975; Peterka and Benolken 1995; Loughlin and Redfern 2001; Redfern et al. 2007; Cruz et al. 2018) and virtual (Dijkstra et al. 1994; Kuno et al. 1999; Keshner and Kenyon 2000; Oie et al. 2002; Sparto et al. 2004; Musolino et al. 2006; Scholz et al. 2012; Hanssens et al. 2013) stimulation. Especially with stimulation at 0.2 Hz, subjects exhibited a steady-state response in the trajectory of their COP containing high power in the frequency regime of the stimulus, which is estimated to be around the mean eigenfrequency of the postural system (Dijkstra et al. 1994). Responses to frequencies above about 0.3–0.5 Hz have been shown to decline strongly in power (Lestienne et al. 1977; van Asten et al. 1988b). Thus, the stimulus in most previous studies has not exceeded these frequencies. A few studies which did use higher frequencies in their stimuli confirmed expected weak responses (e.g. Oida et al. 1995; Peterka 2002). In agreement with these findings, our stimulation at 1.5 Hz elicited responses about five times lower in power than stimulation at 0.2 Hz (Figs. 4 and 5). Weaker responses can originate firstly due to biomechanical constraints that prevent the entire human body to sway at the higher frequency, if modeled as a one-segment inverted pendulum around the ankle joint (Jeka et al. 1998; Peterka 2002). Secondly, if the amplitude across frequencies is held constant, as it was in our study, visual motion at higher frequencies automatically implies higher velocities of the visual scene, i.e. optic flow on the retina. When these velocities exceed a threshold, the illusory percept of self-motion decreases, as the visual system no longer attributes the movement to self-motion (Jeka et al. 1998; Kuno et al. 1999; Barela et al. 2009; Dokka et al. 2010; Day et al. 2016). Similar saturation occurs when the amplitude is increased, while keeping the frequency constant, which also increases optic flow velocity (Dijkstra et al 1994; Peterka 2002).

Our subjects exhibited responses even to visual stimulation at 1.5 Hz, which were small in power (Fig. 4b), but still significant in the time-averaged power spectra (Fig. 5b). Moreover, responses to the high-frequency visual stimulus were highly prominent in the inter-trial phase coherence in both the time-resolved and global spectra (Figs. 4d and 5d). The presence of a response at this high frequency could be explained by more recent models viewing the human body as a multi-link-pendulum (Hsu et al. 2007; Dokka et al. 2009; Scholz et al. 2012; Reimann and Schöner 2017). According to Creath et al. (2005), this includes co-existing excitable modes attributed to various joints of the body. These entail varying amounts of power, depending on biomechanical, environmental and task constraints. Thus, there are postural strategies present during upright standing which respond to high-frequency oscillations. ITPC analysis seems to reliably detect these low-power responses in the trajectory of the COP. This calls for further experiments that use an analogous paradigm with comparably high frequencies, where not only COP but further body segments are tracked simultaneously.

The stimulus was the only temporal reference across trials. Thus, phase coherence can be interpreted as a response coupled, and therefore causally linked, to the respective stimulus. Even when subjects showed comparably weak power at the stimulated frequency, they nevertheless had a strong phase coherence. This discrepancy between power and phase coherence was especially present in trials with high frequency stimulation (Figs. 4b, d and 5b, d). Even when the COP swayed considerably little, it seems to have phase-locked to the visual stimulation in a manner that was highly stable across trials.

In addition, it is noteworthy to mention that in all cases we analyzed raw, unfiltered COP data. As can be seen in the responses to the stimulation at 1.5 Hz, the prevalent low-frequency noise present in the wavelet power spectra disappeared when the data underwent phase coherence analysis (Figs. 4d, 5d). This low-frequency noise most likely reflects the frequency content of unperturbed quiet stance (van Asten et al. 1988b; Singh et al. 2012; Yamamoto et al. 2015) and does not show in the ITPC spectra. In this regard, ITPC seems to represent a filter for coupling of postural sway to a visual drive. Even though the experimental design started each trial at the same phase of the oscillation, the phase coherence between trials was stable throughout the 45 s of visual motion, which is equivalent to at least nine cycles of the oscillation at the lowest presented frequency of 0.2 Hz. This is why an effect of motion onset can be considered neglectable. In addition, during quiet stance, humans exhibit naturally occurring body sway, a stochastic process containing a range of frequencies (Maurer and Peterka 2005; Yamamoto et al. 2015), which means that even when the stimulus started at the same phase in each trial, the subject’s current postural state at motion onset was random. This marks another reason why the constant phase onset was unlikely to be a prevalent factor for the stable phase coherence, but that it must have been due to a coupling of COP to the constant sinusoidal visual input. If COP as being related to COM is considered the main variable controlled by the central nervous system (CNS) to maintain balance, this phase locking represents a dynamic equilibrium (Horak and Macpherson 1996).

A high inter-subject variability regarding frequency power has repeatedly been reported in the literature (Dijkstra et al. 1994; Kay and Warren 2001; Sparto et al. 2004; Mahboobin et al. 2005). All in all, comparison of the two methods used in our analyses proved ITPC to be a more stable measure regarding inter-subject variabilty, and even across frequency (Figs. 6 and 7). To enhance understanding of how balance control is achieved and structured within the human body, it would be of interest to know whether other segments of the body exhibited a phase coherence between trials. As opposed to COM/COP as global variable, this could reveal further insight into possible synergies and multi-joint coordination (Latash 2014; Reimann and Schöner 2017).

**Fig. 10:**
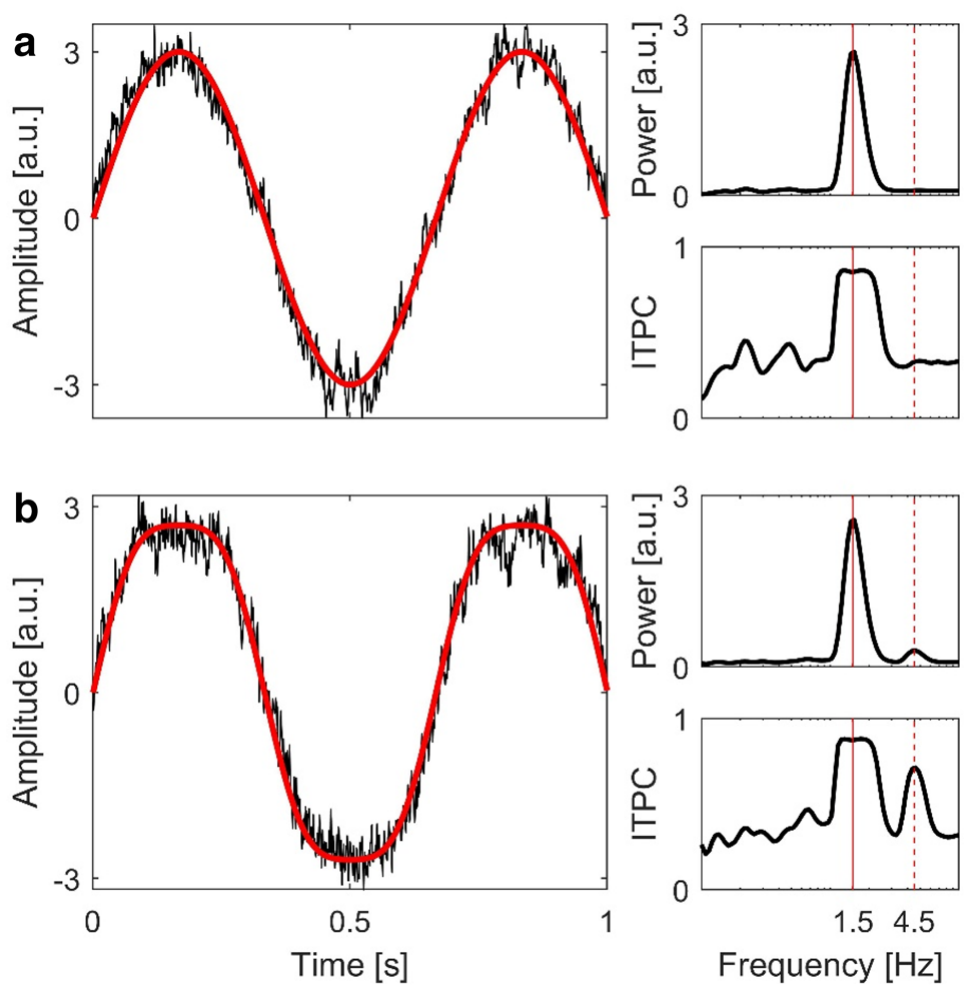
Upper panel **a**: excerpt of a simulated sinewave at 1.5 Hz (red curve) and added pink-noise (black curve). Lower panel **b**: excerpt of a simulated sinewave consisting of oscillatory components at 1.5 Hz and 4.5 Hz with an amplitude ratio of 10:1 (red curve), including added pink-noise (black curve). Panels to the right show time-averaged wavelet power and ITPC spectra of the whole simulated sinewaves (including noise), respectively. Red solid lines represent the main frequency component at 1.5 Hz. Red dashed lines represent the superimposed oscillatory component at 4.5 Hz

The second peak occurring in the ITPC spectrum of the COP when stimulated with 1.5 Hz, which was present at the single subject level as well as in the group data (Figs. 2, 3, 4, 5d), marks another intriguing finding of our study. Within the precision of wavelet analysis, inspection of data from all subjects confirmed it to be triple the frequency of visual stimulation. The synchronicity at this unexpected high frequency can be explained by a harmonic component in the response of subjects’ COP. The observed body sway was not perfectly sinusoidal, in contrast to the predictions of a single-link inverted-pendulum (Winter 1995). More specifially, the observed COP trajectory was flattened at the turning points as compared to a pure sinusoidal oscillation of that frequency (Fig. 10). This could be attributed to internal dynamics in sensorimotor processing and action control, as well as biomechanical constraints such as inertia and time needed for generation of muscle torque. Given the predictability of our stimulus, subjects might have learned the movements pattern after a few cycles and adjusted their postural response accordingly. At the same time, turning points of an oscillation are those parts of the trajectory requiring the largest counteracting acceleration and thereby force. Subjects simply might not have been able to excert that force. Remarkably, wavelet analysis revealed an additional high frequency component in subjects’ body sway. This component was more evident in the coherence analysis, due to its phase synchronicity with the general response, resulting in the strong, well observable peak (Fig. 5d). In addition, and in line with this explanation, the high frequency component seems to increase with increasing frequency.

To simulate such a flattened out sinusoidal, we created a sinewave at 1.5 Hz superimposed by another sinewave at 4.5 Hz with an amplitude ratio of 10:1. We added randomized pink-noise and ran 20 simulations (i.e. trials), on which we performed our wavelet power and ITPC analyses. The simulated signals had the same length (45 s) and sample rate (50 Hz) as our collected data. The results of the simulations are shown in Fig. 10. Superposition of the two sinewaves yielded the predicted flattened out oscillation (Fig. 10b, red curve) when compared to a pure sinusoidal oscillation (Fig. 10a, red curve). ITPC of the superimposed signals yielded spectra highly similar to those obtained by our real data (Figs. 5d, 10b). Accordingly, adding a postural sway component of triple the stimulus frequency in a phase contingent manner results in the observed COP trajectory. We assume that subjects did not add this component intentionally. Instead, it appears automatically. If this reflexive response is only due to biomechanical constraints or aspects of sensory-motor processing like response delay etc., cannot be answered from our dataset. Instead, further studies are required to answer this intriguing question.

Across subjects, our analysis was based on two different stimulus frequencies, both at the far ends of the spectrum of frequencies to be considered for human postural sway. It needs to be further investigated in which range of frequencies inter-trial phase coherence remains a stable measure for postural responses, for COP as well as for additional body segments. Even though additional frequencies have been employed in the experiment, and analysis of those confirmed our general findings, these frequencies were not the same across subjects and thus could not be included in our group analysis. As mentioned above, investigation of the additional frequencies suggests an increase in ITPC of the second peak with increasing frequency. However, further experiments are required to strengthen this point.

We tested the responses resulting from both methods of analysis according to their significance based on a bootstrapped background which we created for each subject (see “Methods”). As the remaining three frequencies presented to the subjects were determined individually, each resulting background was biased towards responses to visual stimulations at different frequencies (Fig. 3). However, in most cases, the remaining frequencies used for the null distribution were sufficiently far from the two frequencies investigated. In addition, averaging over subjects diminished these individual biases (Fig. 5). As mentioned in our Methods section, an actual baseline in the form of a separate experimental condition would be preferable in future studies. If we were to investigate into ITPC only, an ideal baseline condition consisted of each individual sphere oscillating in the anterior–posterior direction at the same frequency, but with a different phase onset, thus not allowing for coherent coupling to an omnipresent phase. This approach would provide us with a justified and unbiased background, which is comparable across subjects.

We preferred to use wavelet-based frequency spectra for our subsequent ITPC analysis over classic FFT decomposition as described in our methods (Data analysis) and confirmed by our findings (Results based on classic FFT decomposition). The most apparent advantage of using wavelets is their additional temporal resolution, which allows for insight into how the spectra evolve over time. This is particularly interesting, and even necessary, for the newly introduced ITPC when using more complex paradigms in which stimulation is not constant over time (e.g. a single stimulus frequency during a whole trial) but rather includes frequency and phase shifts. In addition, even in the context of our time-invariant paradigm, FFT decomposition of the signals led to the predicted artifacts in ITPC, in particular at the single-subject level, where results were hardly interpretable (Fig. 8c, d). A constantly high ITPC resulting from slow drifts in the signal might overshadow actual responses at lower frequencies. At higher frequencies, the ITPC background consisted of substantial noise covering the whole range of possible ITPC values, resulting from a myriad of high-frequency sinewaves the FFT required in order to fit the data. The wavelet spectra were better behaved and all peaks in the power and ITPC spectra based on wavelet decomposition yielded significant results when tested for multiple comparisons. Taken together, these factors favor wavelet-based frequency decomposition over a rather classical Fourier transform when performing subsequent ITPC analysis.

## Conclusion

In our study, we investigated the influence of oscillatory visual perturbations on postural sway by measuring COP trajectories and analyzing frequency content according to established and novel methods. In addition, we introduced a bootstrapping approach to identify statistically significant coupling of body sway and the stimulus. The stimuli presented in VR elicited postural responses in almost all subjects at each of the presented frequencies. Even when responses at the respective frequency bands were weak, testing against the bootstrapped background showed them to be significantly correlated with the stimulus. ITPC revealed coupling to stimulation even at considerably high frequencies above 1 Hz. In addition, coupling to the visual drive in a second frequency band emerged, at about three times the frequency of stimulation. All in all, inter-trial phase coherence proved to be a reliable measure to investigate coupling of COP to oscillatory visual drive, potentially allowing for novel insight in the field of human visuomotor control.
